# Impact of COVID-19 on the Organization of Cancer Care in Belgium: Lessons Learned for the (Post-)Pandemic Future

**DOI:** 10.3390/ijerph191912456

**Published:** 2022-09-30

**Authors:** Ilyse Kenis, Sofie Theys, Ella Hermie, Veerle Foulon, Ann Van Hecke

**Affiliations:** 1 Clinical Pharmacology and Pharmacotherapy, Department of Pharmaceutical and Pharmacological Sciences, KU Leuven, 3000 Leuven, Belgium; 2 University Centre for Nursing and Midwifery, Department of Public Health and Primary Care, Ghent University, 9000 Ghent, Belgium; 3 Science in Nursing and Midwifery, Faculty of Medicine and Health Sciences, Ghent University, 9000 Ghent, Belgium; 4 Nursing Department, Ghent University Hospital, 9000 Ghent, Belgium

**Keywords:** oncology, cancer, COVID-19, coronavirus, patient-centered care, change management, organizational change, quality of care

## Abstract

The COVID-19 pandemic has posed tremendous challenges to healthcare systems. Care for oncology patients, a vulnerable population during the pandemic, was disrupted and drastically changed. A multicenter qualitative study was conducted in 11 Belgian hospitals with the aim to provide an overview of the most important changes that were made in the care of oncology patients in Belgium. In each hospital, a nurse or physician was interviewed by telephone. Two rounds of structured interviews—during the first and second waves of the pandemic—were conducted. The data were analyzed using content analysis. The impact of COVID-19 on care practices for patients with cancer was enormous during the first wave. Major changes, including good but also less patient-centered practices, were implemented with unprecedented speed. After the initial wave, regular care was resumed and only limited new care practices were maintained. In only a few hospitals, healthcare teams reflected on lessons learned and on the maintenance of good practices that came from the COVID-19 experience. As a result, opportunities for healthcare innovation and quality improvement seemed to be missed. Our recommendations aim to support policymakers, hospital managers, and healthcare professionals to learn from the COVID-19 pandemic and to drive patient-centered initiatives in future cancer care.

## 1. Introduction

The outbreak of the Coronavirus Disease 2019 (COVID-19), followed by the strict rules and protective measures taken by national governments, has posed new and tremendous challenges to healthcare systems. COVID-19 and the associated measures led to significant changes in (the organization of) care and impacted the management of noncommunicable diseases [1]. Worldwide, health services were partially or completely disrupted, healthcare staff was reassigned to support COVID-19 services, and screening programs were postponed. Therefore, many patients with noncommunicable diseases have not been able to receive the health services they needed during the pandemic [1].

This is especially relevant for oncology patients as continuity of cancer care is needed, and timely diagnosis and treatment are crucial to avoid disease progression [2]. Moreover, oncology patients are a high-risk group in the COVID-19 pandemic as their immunosuppressed status may cause an increased risk of infection [3,4]. Additionally, when infected with COVID-19, oncological patients have a poorer prognosis as they are at increased risk of serious complications, such as intensive care admission or even death [5,6]. In Belgium, a significant excess mortality of about 400 deaths in patients with cancer was observed in April 2021, corresponding to a 33% increase in the mortality rate [7].

However, despite this, cancer care during the COVID-19 pandemic was fragmented. Due to restrictive measures to minimize the risk of exposure of oncology patients to COVID-19, and due to the re-deployment of resources and workforce to COVID-19-related care, cancer care services were often discontinued or delayed [8]. Healthcare professionals (HCPs) decided to cancel or postpone planned operations, oncological treatments, and consultations to keep patients away from the potentially hazardous environment of the hospital [9]. Moreover, interruptions or changes in the care process were also initiated by patients themselves. Patients seem to have canceled or postponed their consultation or examination in the hospital for various reasons, such as fear of infection [10,11].

During the first period of the pandemic, decisions regarding health care were often made based on early, small reports. Later, several initiatives for recommendations and guidelines were undertaken to support HCPs. The aim was to guarantee well-considered and uniform decisions across hospitals [12]. However, these guidelines were mainly focused on medical/surgical treatments and did not address other aspects of daily clinical practice in oncology (e.g., consultations, follow-up care, and psychosocial care). Consequently, hospital management and HCPs had to make unguided decisions regarding multiple aspects of the organization of cancer care.

Although the COVID-19 pandemic was—to say the least—a challenge for the healthcare sector, the pandemic and the associated reorganization of cancer care offers an opportunity for policymakers, management staff, and HCPs to reflect, learn and become inspired for future (post-)pandemic care. Not only should cancer care be restored, but it should also be improved by taking into account the lessons learned from this pandemic.

Therefore, an overview is needed of which changes were implemented in oncology practice and how HCPs perceived these changes. In this qualitative study, we have obtained an overview of the most important changes that were made in the care of oncological patients in Belgium due to COVID-19. More importantly, the findings will be discussed in light of other research regarding COVID-19 experiences of HCPs, patients, and their relatives. Lessons learned and recommendations will be formulated to improve the quality of cancer care in the long term and to be more prepared for future crises.

## 2. Materials and Methods

### 2.1. Aim

The aim of this descriptive qualitative study is (1) to give a concise overview of changes in the organization of care for patients with cancer in Belgium due to COVID-19, and (2) to thoroughly discuss learnings from the COVID-19 experience for (post-pandemic) cancer care based on the findings.

### 2.2. Design

A multicenter qualitative study was conducted in 11 hospitals in Flanders, Belgium. The study had a descriptive design. COVID-19-related changes in care (organization) of patients receiving non-surgical cancer treatment were inventoried by structured telephone interviews with HCPs. The interviews were analyzed using content analysis [13]. Next, the findings were discussed in light of other studies about (COVID-19-related) changes in care, and necessary considerations for future care were made.

### 2.3. Population and Setting

All included hospitals participated in an implementation research project with the aim to optimize current care processes for patients treated with oral anticancer drugs (OACD) by implementing a care pathway. This project is called the Collaborative Network to Take Responsibility for oral AntiCancer Therapy (CONTACT). The 11 participating hospitals included two university hospitals; the remaining were general hospitals. In one university hospital, two different oncology departments (hematology and digestive oncology) were included, while in the other ten hospitals, only one department participated. One HCP (nurse or physician) from each department, who was sufficiently involved in the care of patients with cancer, was interviewed. Afterwards, a second HCP was consulted to validate the data obtained from the first interview.

### 2.4. Data Collection

Two rounds of structured interviews were conducted: (a) in April–May 2020, during the first wave (10 March–21 June 2020) and (b) in November–December 2020, during the second wave of the COVID-19 pandemic in Belgium (31 August–undefined) [14].

A nurse or physician from each hospital was contacted by telephone and asked questions on the impact of COVID-19 on care practices for patients with cancer. A purposeful sampling technique was applied to select HCPs who were sufficiently involved in the care of patients with cancer and could provide us with the necessary information on the implemented changes due to COVID-19. An interview guide was used focusing on different aspects of the care process, including new and ongoing treatments, hospital consultations, follow-up examinations, involvement of primary care, and psychosocial care. The interview guide can be found in the Supplementary Materials (Table S1). Based on intermediate analysis, the topics of teleconsultations and general safety measures were added to the interview guide as these seemed important changes in the current organization of care. During the interviews, we asked the participants to describe concrete examples of implemented changes (e.g., an example of when a treatment was postponed) to verify and obtain a more objective view of the data. Additionally, member checking was applied verbally during the interviews.

All interviews were conducted by the first author (IK), who was familiar with the organization of oncological care in the participating centers. The interviews were audio-recorded. During the interview, extensive notes were taken. After the first round of interviews, a summary of the changes and modifications in care practices per hospital was made based on the notes and the audiotapes.

After six months, HCPs were interviewed a second time to investigate how the (organization of) care in the hospitals during the second wave differed from care provided during the first wave. If possible, the same HCP was interviewed. If not, another HCP with sufficient knowledge of the organization of cancer care was interviewed. First, the interviewer and HCP went through the summary document—obtained after the first interview—and the HCP was asked to indicate which modifications were retained and which were not. In this way, additional changes in care organization or resumption of normal practices were identified. Through additional questions, information on the period between the two waves or the “inter-wave period” (22 June and 30 August 2020) was collected [14]. Last, the HCP was asked to reflect on the organization of cancer care after the pandemic, and what modifications/new practices they would prefer to keep or not. At the end of both rounds of interviews, thematic saturation was reached.

### 2.5. Data Analysis

A qualitative content analysis of structured interviews with HCPs was conducted. The analysis was descriptive as the authors aimed to stay as close as possible to the data when analyzing, summarizing, and interpreting to obtain an objective overview of the most important changes in cancer care. An inductive approach was applied to ensure that all changes, including unexpected or exceptional changes, were captured.

Per hospital, the first author (IK) made a summary document in Microsoft Word, based on the extensive notes taken during the interview. Audiotapes were re-listened to complete the summary when information was missing or unclear. To structure the summary, the themes of the final interview guide were used, i.e., the aspects of the care process (new and ongoing treatments, on-site consultations, teleconsultations, safety measures, psychosocial care, and primary care). To further shape and complete the summary, questions from the interview guide were used as subthemes. Given the highly structured format of the interviews, this was a convenient and efficient method to obtain a clear summary document per hospital, showing the main changes for each aspect of care. In the Supplementary Materials (Table S2), an example of a summary document can be found.

The summary documents were read by two other members of the research team (AVH, VF) at the end of each round of interviews, followed by a group discussion. Following their comments and critical reflections, the first author verified certain data and/or added data to complete the summary document. Thereafter, the summary document was presented to an additional physician or nurse of the participating hospitals (different than the one interviewed) to ensure the accuracy and completeness of the information. Finally, all summary documents of the two rounds of interviews were inductively analyzed by two authors (IK, EH) separately. Based on the summary documents of each department, the authors (IK, EH) summarized the most important changes for each of the aspects of care that were questioned in the interviews. Both summaries were compared and discussed by the research team in order to obtain an objective and representative overview of the main changes across the included hospitals.

## 3. Results

Twelve hospital departments participated in the first round of interviews. Of the HCPs interviewed, four were oncologists, seven were nurses, and one was a head nurse. Two hospital departments dropped out in the second round. In two other hospital departments, the interviewed HCP was no longer available for a second interview. Therefore, a colleague was interviewed instead. In total, eight nurses, one head nurse, and one oncologist were interviewed in the second round. The characteristics of the participating HCPs can be found in Table 1.

**Figure 1:**
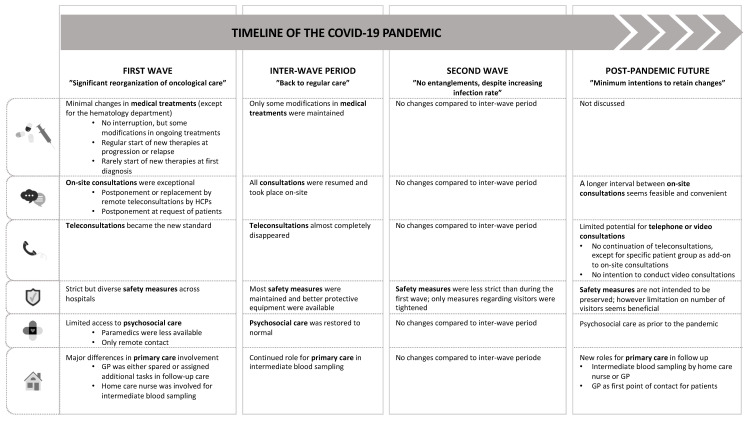
Overview of changes in the organization of oncological care over time and future prospects.

The results below are ordered by time period (first wave, inter-wave period, and second wave) and by aspect of care. Figure 1 gives an overview of the most important changes in care organization over time and of future prospects, and thereby summarizes the most important results.

### 3.1. First Wave: Significant Reorganization of Oncological Care

#### 3.1.1. Minimal Changes in Medical Treatment

In all hospitals, except for the one hematology department that participated in the study, ongoing cancer therapies were not interrupted. Only in exceptional cases or at the request of the patient, HCPs decided to interrupt a treatment, e.g., in the case of difficult telephone follow-up, at a patient’s request, or in the treatment of elderly patients. At the hematology department, ongoing therapies were paused when possible.

Mostly, ongoing therapies remained unchanged. However, for some patients, treatments were modified with the aim to minimize the risk of infection with COVID-19. These decisions were based on guidelines provided by (inter)national professional organizations for oncology and hematology to support HCPs in dealing with the COVID-19 pandemic [12,15,16,17]. However, they strongly focused on medical aspects and paid much less attention to psychosocial aspects. In addition, physicians considered individual patient characteristics when making a decision. 

New therapies were started as usual. In only two hospitals, the start of new therapies was temporarily delayed whenever possible considering the patient’s characteristics (age, comorbidities) and disease status. For example, in the case of older, vulnerable patients with a lot of comorbidities and with a relatively stable disease, the treatment was postponed for a few weeks. In the hematology department, stem cell transplant was only conducted in most critical, acute cases. In two hospitals, the choice of the new treatment was influenced by the COVID-19 pandemic, for example, by avoiding therapies associated with a risk of long-term neutropenia when defining a new therapy plan. However, HCPs indicated that the number of new diagnoses was very limited since the start of the COVID-19 pandemic.

#### 3.1.2. On-Site Consultations Were Exceptional

For each patient, physicians individually decided whether to have the consultation in the hospital or by telephone. In only one hospital, consultations were continued as usual, while in the other hospitals only a limited number of consultations took place in the hospital. Only consultations related to in-hospital treatment, very urgent consultations, or consultations regarding a new diagnosis were continued on-site. Almost all follow-up consultations were postponed or replaced by a telephone consultation. For patients treated with OACD, the interval between the physical consultations in the hospital was extended from four to eight weeks. In the fourth week, a blood sample was taken at home (by a home nurse or general practitioner (GP)), followed by a telephone consultation with the physician to discuss the results. At week eight, patients had to go to the hospital for medication refill at the hospital pharmacy, which was combined with a physical consultation. Not only consultations with the physician, but also nursing consultations, no longer took place in the hospital but were replaced by telephone consultations.

The postponement of a consultation or the switch to teleconsult was usually communicated to the patient by the medical secretariat by telephone, and only occasionally by the attending physician or the supervising nurse.

At the beginning of the COVID-19 pandemic, patients themselves frequently asked for postponement of their consultation because they felt it was not safe to go to the hospital. Usually, the physician assessed the individual request to evaluate if the consultation could be postponed or replaced by a telephone consultation. If the physician considered a physical consultation necessary, efforts were made to convince the patient of the importance of the consultation. At the time the interviews were conducted, patients were already less inclined to ask for postponement than during the first weeks of the pandemic because they were reassured by the safety measures taken by the hospital.

#### 3.1.3. Telephone Consultations Became the New Standard

Telephone consultations were usually performed by the physician or less frequently by the ONN or APN. Prior to the COVID-19 pandemic, some ONNs or APNs already performed telephone consultations. The number of teleconsults, however, increased enormously during the pandemic. Depending on the purpose and the content of the consultation, the physician opted for a telephone or on-site consultation, e.g., when a teleconsult did not seem appropriate for discussions regarding a new diagnosis, progression or negative test results. When HCPs were asked how they thought these telephone consultations were experienced by patients, they told that most patients were not opposed to those telephone consultations. HCPs indicated that, often, patients even seemed to feel relieved that they did not had to go to the hospital and could thus minimize the risk of infection. Nevertheless, most HCPs were not fond of teleconsults due to the following disadvantages: the reduced possibility to provide psychosocial support, more difficulties discussing certain (complex or delicate) topics, the inability to conduct a clinical examination, and shorter and more superficial conversations. In addition, certain problems (e.g., the occurrence of side effects) seem to go unnoticed and patients are less likely to disclose these problems in a telephone conversation. Video consultations can be a partial solution to this problem, although it also brings difficulties. Due to technical problems and challenges in the elderly population who are not familiar with the technology, video calls were only performed in 2 of the 11 hospitals.

#### 3.1.4. Strict but Diverse Safety Measures across Hospitals

To prevent the spread of the coronavirus, safety measures were taken in each hospital. These measures differed strongly between hospitals and were usually imposed by the local hospital management. The most common measures were related to hand disinfection, social distancing, wearing a face mask (HCPs, and/or patients and their attendants), temperature measurement at the hospital entrance, restriction of the presence of attendants during consultations, ban on visitors during (one-day) hospitalizations, limited waiting room capacity, COVID-19 testing, and/or telephone triage questionnaires prior to hospitalization. Hospital policies differed mainly in: who was obliged to wear a mask (HCPs and/or patients and their attendant), which masks were allowed to be worn by patients and attendants (own mask/mask provided at hospital entrance), the number of attendants allowed during consultations, and the application of testing or triage prior to hospitalization.

Prior to admission for (one-day) hospitalizations, patients were screened for COVID-19 by using a PCR test and/or a telephone triage questionnaire. In the case of a COVID-19 infection or increased risk of infection, treatment was postponed, or very strict precautions were taken. However, based on the interviews, there seemed to be only a few cases of infection. When postponement of treatment was not necessary or not possible, infected patients were admitted but had to take a different route through the hospital than the other patients so that both “circuits” were completely separated, and contact was avoided. Additionally, extra strict protective measures were applied (e.g., a face shield was used, or clothes were changed).

#### 3.1.5. Limited Access to Psychosocial Care

The involvement of other HCPs—such as a psychologist, social worker, and dietician—in the care of patients with cancer changed during the pandemic. In most hospitals, physical consultations by the psychologist, social worker or dietician were no longer possible. However, they were usually available by phone when needed. Consequently, patients with cancer only had limited access to psychosocial care. Moreover, two HCPs indicated that the psychologist was mainly at the service of the HCPs and as a result became less involved in the care patients with cancer. Only one HCP told that, due to the increased need for psychological support during the COVID-19 pandemic, the psychologist was more involved than prior to the pandemic.

#### 3.1.6. Major Differences in Primary Care Involvement

We saw large differences in the involvement of the GP during the pandemic. Depending on the hospital, GPs were either more or less involved in the care of patients with cancer. Some HCPs also indicated that there was no difference in GP involvement compared to the pre-pandemic situation. If GPs were less involved, the main reason was to relieve them of the increased workload and burden they experienced due to COVID-19. However, in some hospitals, the GP was more intensively involved in patient follow-up to reduce or avoid hospital visits. Hospital staff clearly appointed the GP as the first point of contact for patients in case of problems or complaints. GPs were also engaged in intermediate clinical assessment, medication administration, and blood sampling. The latter was also performed by home care nurses. In several hospitals, there was an increased involvement of home care nurses specifically for intermediate blood sampling. The HCPs mentioned that most patients seemed very satisfied with this new way of working because they had to travel to the hospital less frequently and waiting time in the hospital could be reduced.

### 3.2. Inter-Wave Period: Back to Regular Care

In all hospitals, regular care was (almost completely) resumed between early May and early June 2020. Most changes introduced in response to the COVID-19 pandemic were not maintained in the inter-wave period.

Treatments modified during the first wave were often switched to the original therapy plan again. However, some treatment modifications were maintained. Examples include an extended interval between administrations of chemotherapy/immunotherapy, more administration of growth factors, and switch in treatment order (first treatment with hormone therapy followed by other treatments such as surgery or chemotherapy). It is important to note that there were significant treatment variations between hospitals, with hospitals completely returning to pre-COVID-19 treatment protocols, while others retained some changes. Only in the participating hematology department, treatments were still postponed whenever possible because of the risk of overcrowding in the intensive care unit.

Most consultations were resumed as they were prior to the pandemic, i.e., in the hospital. Telephone consultations were only conducted in a few exceptional cases, e.g., at patient request, and in those hospitals where telephone consultations were already current practice prior to the first wave.

During the inter-wave period, a number of safety measures were maintained to avoid the spread of and infection with the coronavirus. The measures in place in most hospitals were social distancing, only one attendant during consultations, and no visits during one-day hospitalizations. Screening took place prior to admission for (one-day) hospitalizations, while in some hospitals, screening was no longer performed. When regular care was resumed, additional safety measures were taken for HCPs such as use of safety goggles, face shields and/or FFP2 masks as these protective materials were now available, unlike during the first wave.

Psychologists, social workers, and dieticians were available again, also for on-site consultations. Therefore, access to psychosocial care was restored. The hospital oncology teams continued to call on primary care professionals—the GP and home care nurse—for intermediate blood sampling.

### 3.3. Second Wave: No Entanglements despite Increasing Infection Rate

In most hospitals, no changes occurred in the organization of care during the second wave compared to the inter-wave period. Only in a few hospitals, restrictive measures regarding the attendance of relatives during consultations and visits during (one-day) hospitalizations were tightened again. Safety measures were thus less restrictive during the second wave than during the first wave of the pandemic. Reasons that were mentioned most frequently for these differences in organization of care between the first and second wave were: measures applied during the first wave did not respect the rights and humanity of patients, HCPs were better prepared to deal with the situation because COVID-19 was no longer unknown, less anxiety and panic among patients, and telephone contacts were found to be inefficient and impersonal by HCPs. Last, the hospital management also imposed looser measures compared to the first wave, leaving more room for hospital staff to freely decide on the restrictions applied.

### 3.4. Post-Pandemic Future: Minimum Intentions to Retain Changes

In many hospitals, post-pandemic future prospects for the organization of care for patients with cancer (which changes to discard and which to retain) were not yet discussed. Only a few HCPs indicated that certain changes in the organization of care as a result of the COVID-19 pandemic were considered beneficial for patients and/or HCPs and would be continued in the future. A beneficial change that was most often mentioned was the intermediate blood sampling by a GP or home care nurse. In one hospital, they agreed to continue to emphasize the role of the GP as the first point of contact for patients, also after the pandemic has past, as this would reduce the workload for the HCPs in the hospital. In many hospitals, HCPs seemed to appreciate the peace and quiet in the outpatient department that was created by limiting the number of visitors. Some of the HCPs were convinced that this was also beneficial for patients. They would therefore prefer to keep limiting or prohibiting visits during one-day hospitalizations for chemotherapy. Last, at two hospitals, they planned to maintain the longer interval between physical consultations in the hospital for patients on OACD, at least for those with stable disease.

Most HCPs agreed that teleconsultations will no longer be a systematic part of the care process because of the reasons mentioned above. Some HCPs saw potential in the use of telehealth in the future but only for a limited group of patients, e.g., in addition to regular physical consultations in the hospital for patients who need more intensive follow-up. However, they were convinced that it is not suitable/needed for every patient and in every situation. Due to the difficulties in using video consultations, they were not frequently used during the pandemic and there was no interest in implementing this in the future.

## 4. Discussion

This qualitative study provides insight into the impact of COVID-19 on care practices for patients with cancer during the early period of the pandemic in Belgium. We will discuss these findings in depth, and we will draw some lessons from this multicenter COVID-19 experience for the (post-pandemic) future.

### 4.1. A Need for Guidance

During the first wave, major changes were made to the care process for patients with cancer. The main reason for this reorganization of care was to minimize the risk of infection for these patients, who were considered a more vulnerable population in the COVID-19 pandemic. Decisions regarding medical treatment were based on early guidelines provided by (inter)national oncology and hematology organizations [12,15,16,17]. However, guidelines regarding other aspects of care were not available. Hospitals were left in the dark and felt uncertain about how they were handling this unprecedented situation. Several HCPs who participated in this study therefore asked our research team to share an overview of the implemented changes in other hospitals. After consent and validation by a second person, we bundled the obtained overview documents per hospital in one booklet “learning by sharing best practices”, which was shared among all participating hospitals. Sharing this information seemed to help the HCPs to learn from each other and also to become more confident about their own (changed) practices. Creating a learning community can be useful in a future crisis situation or in general to intensify the communication and collaboration between hospitals. Additionally, future HCPs should be (better) prepared and trained to deal with such crisis situations. Training should aim at making HCPs more resilient and better ‘armed’ in case of future crises.

Due to a lack of guidance, the applied changes largely differed between settings. Albeit, in general, the impact was enormous. Patients could no longer see their HCP in the hospital, contact with HCPs was carried completely by phone, the psychologist and social worker were not available, relatives were not allowed to visit or accompany patients in the hospital, etc. Roadmaps for future pandemics or other crisis situations must be available and cover all aspects of care to ensure more uniform care changes that can be tailored to the local context, and to avoid low-quality care. However, additionally, decisions should be made on a patient-by-patient basis and individual characteristics and preferences should be taken into account [9]. The individual behind the patient might not be forgotten in times of crisis. Therefore, patient-centeredness should be emphasized in the guidelines.

### 4.2. Where Is Patient-Centered Care?

Although decisions during the first wave were made by policymakers, management staff, and HCPs with the intention to protect patients, these changes also had an important downside and caused serious harm to the well-being of patients. Multiple quantitative studies have shown a negative impact of COVID-19 on the well-being of patients with cancer [18]. Increased levels of psychological distress and anxiety were observed. We further explored these findings in a qualitative study, including interviews with patients with cancer and their relatives. We found that it was difficult for patients and relatives to cope with the changes in their care process. Therefore, patient-centered care and communication was proven to be even more important during a pandemic or in other situations in which planned care might be changed [11]. Based on this research, we have developed recommendations on patient-centered counseling and follow-up to guide oncology professionals during the COVID-19 pandemic but also beyond. The study showed that it is important for HCPs to explore the meaning of the change for patients and consider its impact on the patient’s well-being in the decision-making process. Moreover, the results emphasized that access to psychosocial care must be guaranteed at all times. Additionally, the involvement and presence of relatives must be preserved as far as possible. However, in this current study, we see that several reported care practices do not align with these patient-centered recommendations or a patient-centered approach. Provided care was strongly responsive to the pandemic, but lost responsiveness to patients’ needs.

### 4.3. A Change Process with Remarkable Speed

The hospitals included in this qualitative study also participated in an implementation research project on care pathways for patients treated with OACD (CONTACT). In the middle of this project, the hospitals were faced with the COVID-19 pandemic. All of a sudden, daily clinical practice had to be rapidly adapted to anticipate the threat of COVID-19. Major changes were implemented over a significantly short period of time. Evidence-based decisions regarding the organization of health care were made and implemented at a European, national and hospital level by policy makers and hospital management staff. Subsequently, work routines were immediately switched, and new practices were imbedded by HCPs. In contrast to this rapid implementation of COVID-19-related practices, the implementation of new interventions in the CONTACT project was challenging and very time-consuming. The high speed and efficiency of change during the first wave contrast sharply with the slowness and complexity by which change in health care is normally characterized [19,20,21]. In the study of Nilsen et al. (2020), predictability of and preparedness for change emerged as essential conditions for the successful implementation of new interventions. Implementing change is more likely to succeed at a relatively slow pace, as this allows for the preparation and learning throughout the change process [22]. However, these conditions were not applicable at the time of the COVID-19 outbreak.

Although changes were implemented quickly, most changes were only implemented during a short period of time and did not remain integrated in daily practice after the initial wave. How can we explain this rapid “forth and back” movement?

Several authors have investigated how to successfully manage organizational change in health care [22,23,24,25]. Adequate leadership was often identified as an important prerequisite to achieving change in (cancer) care. More specifically, leadership must be shared between the hospital management and the healthcare team. On the one hand, a top-down management approach is required to create commitment and drive for reorganization. On the other hand, a more bottom-up approach and a certain degree of self-directedness by the team are needed for the successful and sustainable implementation of changes [25,26]. During the early period of the pandemic, reorganization was strongly imposed by local management leaders and governances [27,28]. This strict top-down approach enabled fast decision-making and adaptation to COVID-19-related challenges, which are crucial in times of crisis [27]. Especially during the first wave, time was limited. Restrictions needed to be effectuated as soon as possible, leaving no or less room for the involvement of HCPs. However, in the study by Rücker et al. (2021), HCPs emphasized that—even in a crisis—leadership must be supportive. Some included HCPs indicated that decisions were made by management leaders who had little understanding of the current situation at the workplace. Moreover, there was less room for the input of HCPs in the decision-making process. However, there were also participants that described a good supportive leadership style by their management [28]. Although we understand that decisions had to be made and implemented quickly, and the involvement of HCPs was probably not realistic during the first period of the pandemic, sufficient attention must be paid to supportive leadership during the chronic phase of the pandemic. All involved stakeholders should be invited to the table, including representatives of every group of HCPs (e.g., oncology nurses, ONNs, APNs). This shift from a top-down approach in the first wave to a more bottom-up leadership style in the second wave was seen in Dutch home healthcare according to van den Bulck et al. (2022).

The pandemic offered a good learning experience of what good and bad leadership can look like. A continued profound top-down approach, lack of supportive leadership, and the inability of HCPs to influence the change may—next to other factors—explain why some implemented changes that appeared to have some value (e.g., remote teleconsultations for a specific subgroup of patients) were not sustained over the longer term [22,25,28].

Another prerequisite for successful change in health care is that the involved stakeholders recognize the problem and understand the need for change [22,25]. Obviously, HCPs agreed that changes were needed to anticipate the threat of COVID-19. However, during the second wave, the threat diminished and the need for change decreased. Consequently, HCPs no longer saw the value of several new practices (e.g., telephone consultations), and these practices were therefore abandoned. However, some implemented changes (e.g., intermediate blood sampling by home nurses) were preserved because they proved to be beneficial to HCPs and/or patients.

Other conditions that may have influenced the implementation of new practices are the increased team spirit within and across hospitals and the excessive workload during the COVID-19 pandemic. The team spirit was exceptionally high during the early period of the pandemic. Colleagues found comfort and strength in each other, which may have had a positive impact on successfully achieving change [28]. This is in line with the knowledge that a supportive working environment and good communication are key to change management [26]. Team spirit increased not only within one hospital but also between different institutions. Communication was more efficient, and teamwork was stronger due to solidarity between hospitals [29]. Good practices were shared and thus a learning community was created. Joint learning across organizations is known to facilitate the implementation of changes in health care [30,31]. However, the workload was extremely heavy due to additional tasks and workforce problems [27]. Over time, resilience among HCPs decreased. This might explain why HCPs were so eager to return to “normal”.

### 4.4. A Plea Not to Return to Normal

Only minor changes were retained after the first wave passed. In some hospitals, intermediate blood sampling was still performed by the home care nurse, or teleconsultations were still used for a specific subset of the patient population. However, in most cases, HCPs seemed to have only little intention of sustaining changes. The desire to return to the normal pre-pandemic situation was strong. According to the HCPs, the most common reason for leaving new practices was the fact that these practices were not respectful to patients and their relatives. As mentioned before, we agree that several implemented changes were not patient-centered. However, COVID-19 also brought about some positive changes to health care. First, the pandemic has taught us about efficient healthcare leadership, multidisciplinary teamwork, and between-hospital collaboration. Second, the COVID-19 pandemic has exposed critical weaknesses in healthcare systems, and more specifically in current cancer care organizations. The practices and strategies that were implemented to solve problems faced during the pandemic could therefore also be a potential solution for already existing pre-pandemic problems in health care (e.g., workforce shortage) or could be used to increase the quality of health care. For example, increasing the interval between on-site consultations or replacing on-site by remote telephone consultations and task shifting to primary care HCPs could be used to reduce workload. Additionally, teleconsults for additional follow-up, and collaboration with primary care can increase overall care quality. Therefore, health care must not go back to normal. Health care must be pushed to a higher level of quality. The pandemic has reduced barriers to change and enabled healthcare innovation. We should use this momentum to strive for better care, instead of restoring “normal” care.

The interviews revealed that most healthcare teams had not yet discussed the pros and cons of the implemented changes or thought about which changes could potentially be useful in the long term. Therefore, we would like to invite all policy makers, hospital managers and HCPs to reflect on their COVID-19 experience and think about what they have learned and what they can take forward to clinical practice or policy making in a (post-)pandemic future.

With the findings of this study in mind, we have formulated important lessons learned and recommendations, hoping that these can support more patient-centered cancer care in the new normal.

### 4.5. Lessons Learned for a (Post-)Pandemic Future

First of all, we have summarized recommendations to inform care models for future pandemics and other crisis situations based on the experiences of the COVID-19 pandemic.

1. Healthcare teams should be sufficiently supported by guidelines and recommendations. A roadmap for future pandemics or similar situations should be prepared and made available as soon as possible when a crisis arises. A roadmap can provide structure to HCPs in a chaotic situation, can guarantee more uniform decisions across institutions, and prevent low-quality care. The roadmap should not only focus on treatment guidelines but should include all aspects of the care spectrum.
2. In the education and training of HCPs, sufficient attention should be paid to care provision during a pandemic or similar crisis. In this way, HCPs can be better prepared to handle such crisis situations efficiently, without compromising the quality of care. They must learn to set priorities during a crisis, without losing sight of the patient. Additionally, training can make HCPs more resilient in such difficult and demanding circumstances.
3. Care provision should always and everywhere be patient-centered even in crisis situations. Patients and relatives find it difficult to cope with changes in planned care, whether due to a crisis situation or not. Therefore, HCPs should always consider the impact of change on the patient’s well-being. Not only the risk of infection should be taken into account when making decisions, but also the patient’s concerns, needs, and preferences.
4. Access to psychosocial care must be guaranteed at all times, especially during a crisis situation as elevated distress and anxiety levels are common among patients.

Second, we have also learned a lot from the COVID-19 experience that is applicable outside the scope of a pandemic. We have formulated some recommendations for the **“new normal”**. These recommendations encourage the transfer of care to the patient’s home.

1. Telehealth can be useful for a certain subgroup of patients. Telehealth can be used instead of regular follow-up in the hospital for patients with stable disease, or it can be used in addition to regular consultations for patients in need of closer monitoring.
2. Better communication and collaboration with primary care is not a utopia. As proven during the COVID-19 pandemic, the involvement of primary care professionals (home care nurse and GP) is feasible, e.g., for intermediate follow-up/blood sampling.
3. Treatment protocols can be adapted to reduce hospital visits and therefore healthcare costs.

Following these recommendations, the frequency of hospital visits can be reduced for patients with cancer. Of course, such decisions need to be made on an individual basis and need to be discussed with the patient. In between hospital visits, follow-up can be performed by using telehealth or involving primary care professionals. However, at this moment, the financial structure does not encourage a reduction in hospital visits.

This list of recommendations is not limitative. Some HCPs stressed the added value of visitor restrictions, which could continue to be applied in the future. Moreover, some practices such as wearing masks remained present although the infection rate was declining. More in-depth research on underlying reasons for such practices might be relevant.

### 4.6. Strengths and Limitations

The two rounds of interviews were conducted within very narrow time windows—during the first and second wave—in which critical changes in cancer care were observed. We think this is a major strength of this study as we could reduce recall bias and get a very detailed overview of the changes at each phase of the pandemic.

We applied several techniques to increase the trustworthiness of the findings. First, HCPs were asked to provide concrete examples of the implemented changes they mentioned in the interviews. Second, member checking was applied during the interviews by going through all interview notes when a certain aspect of cancer care was fully discussed and asking the HCPs to verify this. Third, after the interview, the summary document was presented to another HCP from the same department as an additional validation procedure. Fourth, investigator triangulation was applied to avoid biased interpretations of the results. At the end of each interview round, the interview summaries were discussed within a multidisciplinary and highly competent group of three researchers with sufficient knowledge of the organization of cancer care in Flanders. Moreover, the interview summaries were analyzed by two members of the research team separately, one with experience in healthcare research and one with experience in clinical work.

There are also some limitations to this study that need to be considered. First, all interviews were performed by the first author to obtain consistency in the interviews across departments, and to be able to question the HCPs more in-depth (taking into account previous interviews). However, this might also have constituted a bias. Investigator triangulation was applied to limit this bias. Second, we chose a purposeful sampling technique to recruit participants, which might impair the generalizability of the results. We do, however, believe that the results are highly transferrable to all Belgian hospitals and other similar healthcare settings, as different types of hospitals (general/university hospital) and different types of oncology departments (medical oncology, hematology, digestive oncology, and respiratory oncology) were included. Additionally, thematic saturation was reached at the end of each interview round. Third, the interviews needed to be conducted in a very short time. Therefore, there was little time to perform intermediate analyses within one interview round. Last, due to the structured format of the interviews, some issues were not discussed in sufficient depth. For example, we were not able to collect (sufficient) data on the persistence of certain practices when the infection rate declined (e.g., visitor restrictions, wearing masks), and we will therefore not be able to give an explanation for this. It might be useful to explore these issues in future research.

## 5. Conclusions

This qualitative study shows the large impact of COVID-19 on care practices for patients with cancer during the first wave of the pandemic. Changes were implemented with unprecedented speed. However, a lot of changes were not compliant with a patient-centered approach. After the initial wave, regular care was resumed, and although the pandemic also brought some good things, only limited new care practices were maintained. Furthermore, HCPs did not reflect on lessons learned from their COVID-19 experience. As a result, opportunities for healthcare innovation and quality improvement were missed. Our recommendations aim to support policymakers, hospital managers, and HCPs to learn from the COVID-19 pandemic and to inspire them for more patient-centered cancer care in the new normal.

## Figures and Tables

**Table 1:** Characteristics of included healthcare professionals.

Characteristics		
	First round	Second round
**Type of HCP**		
Nurse (oncology nurse, ONN, APN)	7	8
Head nurse	1	1
Oncologist	4	1
**Age**		
≤35 years	3	1
36–45 years	2	3
46–55 years	7	6
**Gender**		
Female	11	10
Male	1	0
**Years of experience in oncology**		
≤5 years	2	0
6–15 years	4	3
16–25 years	4	4
26–35 years	2	3
**Type of hospital of employment**		
General	9	7
University	3	3
**Type of oncology department**		
Medical oncology	9	7
Hematology	1	1
Digestive oncology	1	1
Respiratory oncology	1	1

ONN = Oncology nurse navigator; APN = Advanced practice nurse.

## Data Availability

The data presented in this study are available on request from the corresponding author. The data are not publicly available due to privacy reasons.

## Supplementary Materials

The following supporting information can be downloaded at: https://www.mdpi.com/article/10.3390/ijerph191912456/s1, Table S1: Interview guide for structured interviews with HCPs; Table S2: Example of summary document per hospital (after first interview).
