# Supplementary material for: Impact of COVID-19 on the Organization of Cancer Care in Belgium: Lessons Learned for the (Post-)Pandemic Future
Source: Int J Environ Res Public Health. 2022 Sep 30;19(19):12456. doi: 10.3390/ijerph191912456 (PMC9566094; doi:10.3390/ijerph191912456)
Supplement: Supplementary file 1 [file ijerph-19-12456-s001.zip › ijerph-1923714-supplementary.pdf]

Table S1. Interview guide for structured interviews with HCPs.

**Medical treatment**
What changes have been made to medical treatments due to COVID-19?<br>• Are ongoing medical treatments discontinued or modified?<br>• Is the start of new treatments postponed?<br>• Is the choice of a certain new treatment influenced by COVID-19?
**On-site consultations**
What changes have been made to on-site consultations due to COVID-19?<br>• Do consultations take place in the hospital?<br>• For which patients do consultations take place in the hospital?<br>• Are consultations in the hospital postponed/cancelled?<br>• Are consultations postponed/cancelled on the initiative of the hospital team or the patient?<br>• How are these on-site consultations organized?
**Teleconsultations**
How are teleconsultations used in this time of the pandemic? What is changed compared to before the pandemic<br>• Are teleconsultations conducted in your hospital?<br>• For which patients/for which purposes are consultations performed by telephone?<br>• How do you experience these teleconsultations?<br>• How do you think patients experience these teleconsultations?
**Safety measures**
Which safety measures are taken in the hospital?<br>• Social distancing?<br>• Testing?<br>• Triage?<br>• Protective materials?<br>• Other precautions?
**Psychosocial care**
How is psychosocial care provided to patients during this time of the pandemic? What has changed compared to before the pandemic?<br>• How accessible is the psychologist?<br>• How accessible is the social worker?
**Primary care**
How are HCPs from primary care involved during this time of the pandemic? How has their role changed compared to before the pandemic?<br>• How is the role of the GP changed?<br>• How is the role of the community pharmacist changed?<br>• How is the role of the home care nurse changed?
**Additional**
• Are there other changes implemented in the care for oncological patients due to COVID-19?<br>• Do you want to add something?

Table S2. Example of summary document per hospital (after first interview).

**Medical treatment**
What changes have been made to medical treatments due to COVID-19?
• Ongoing treatments were not discontinued<br>• Very exceptionally, a treatment was modified (the decision was made individually per patient). Possible modifications were:<br>o The interval for immunotherapy was extended (depending on the product and in accordance with guidelines).<br>o Growth factors were administered more frequently.<br>o In adjuvant hormonal treatment of breast cancer, treatment with Taxol weekly was started first (instead of EC) because of the smaller risk of myelosuppression.<br>• Other treatments were not modified.<br>• New treatments were started as usual.
**On-site consultations**
What changes have been made to on-site consultations due to COVID-19?
• The physician decides individually for each patient whether a consultation will take place in the hospital, by telephone, or will be postponed.<br>o All consultations for patient on oral anticancer drugs are postponed unless the treatment was very recently started. The consultation is always replaced by a teleconsult.<br>o If the interval of immunotherapy is extended, the interval between on-site consultations is also extended. A teleconsult is conducted instead.<br>o All consultations for patients on chemotherapy continue as usual in the hospital. Every time a patient must come to the hospital to receive chemotherapy, the patient is seen by the physician.<br>o Follow-up consultations are postponed to a later time.<br>This is communicated to the patient by the oncology secretariat.<br>• Initially, patients frequently called to postpone their consultations. In most cases, this was a follow-up consultation. The secretary rescheduled the consultation to a later time. If the secretary doubted whether postponement is a good idea, he/she discuss this with the physician.<br>Patients did never call to postpone their treatment.
**Teleconsultations**
How are teleconsultations used in this time of the pandemic? What is changed compared to before the pandemic?
Follow-up of patients treated with oral anticancer drugs or immunotherapy is conducted by telephone. Teleconsultations are performed by the physician. Both patients and HCPs find this less convenient than on-site consultations. Telephone consultations are much shorter. Things (side effects, non-adherence, alarm signals, …) are more easily missed in a teleconsult.
**Safety measures**
Which safety measures are taken in the hospital?
At consultations/treatments/examinations in the hospital, the following safety measures were applied:<br>• Patients have to wash their hands with disinfectant gel at the entry<br>• Patients have to wear a mask in the hospital<br>• Social distancing is applied in the waiting room

• Initially, relatives of the patient were never allowed to accompany the patient in the hospital. Now, a relative is only allowed to accompany the patient in the hospital: (a) if the presence of the relative is highly necessary (e.g. for a wheelchair user, a non-native speaker); (b) when discussing the results of an important examination; and (c) when starting a new therapy.	
**Psychosocial care**	
How is psychosocial care provided to patients during this time of the pandemic? What has changed compared to before the pandemic?	
• The psychologist and dietician are still involved in the same way/to the same extent for urgent cases. They are present on the ward/in the day clinic. Follow-up consultations with the psychologist or dietician are postponed in agreement with the patient.<br>• The social worker is involved in a different way/to a lesser extent. They work at home several days a week. On these days, they are still available by phone for patients and their relatives.	
**Primary care**	
How are HCPs from primary care involved during this time of the pandemic? How has their role changed compared to before the pandemic?	
• GPs are less engaged. Patients say that it is harder for them to reach their GP because of overcrowding.<br>• Home care nurses are more involved. They are called in for intermediate blood sampling at the patient's home. Prior to the pandemic, blood sampling was always conducted in the hospital. In few cases, blood sampling was performed by the GP. During the pandemic, blood sampling for patients treated with oral anticancer drugs or immunotherapy (who were now less regularly seen in the hospital) was performed by a home care nurse at the patient's home. The patient received the contact details of the organization of home care nurses and could schedule an appointment by telephone. The home care nurse brings the blood sample to the hospital lab, and the sample is analyzed there. The results are then discussed by the physician in a teleconsult.<br>This was perceived very positive by patients and HCPs. This is something they was to keep doing in the future.<br>For patients on chemotherapy, blood sampling is still conducted in the hospital.	
**Additional**	
• Are there other changes implemented in the care for oncological patients due to COVID-19?<br>• Do you want to add something?	
Before the pandemic, patients went to the hospital pharmacy to pick up their medication themselves. During the pandemic, the nurse picks up the medication at the hospital pharmacy and brings it to the patient.	
